# Organization of oversight for integrated control of neglected tropical diseases within Ministries of Health

**DOI:** 10.1371/journal.pntd.0006929

**Published:** 2018-11-21

**Authors:** Claire Standley, Matthew R. Boyce, Anna Klineberg, Gabrielle Essix, Rebecca Katz

**Affiliations:** 1 Georgetown University Medical Center, Center for Global Health Science and Security, Washington, District of Columbia, United States of America; 2 Department of Health Promotion and Behavioral Science, University of Texas School of Public Health, Houston, Texas, United States of America; University of California Berkeley, UNITED STATES

## Abstract

**Background:**

Neglected tropical diseases (NTDs) are communicable diseases that impact approximately 1 billion people, but receive relatively little research, funding, and attention. Many NTDs have similar treatments, epidemiology, and geographic distribution, and as a result, the integration of control efforts can improve accountability, efficiency, and cost-effectiveness of programs. Here, we examine the landscape of efforts towards NTD integration across countries with the highest burden of disease, and review the administrative management of integration in order to identify approaches and pathways for integration.

**Methodology and principal findings:**

We utilized a standardized system to score countries for NTD endemnicity to create a list of 25 countries with the highest overall burden of NTDs. We then conducted a literature review to characterize the NTD control programs in the focus countries. Six countries were selected for key informant interviews to validate literature review results and gather additional data on opportunities and obstacles to NTD integration, from an administrative perspective. The majority of countries included in the study were located in Africa, with the remainder from Asia, North America, and South America. Multiple models and pathways were observed for the integration of NTD programs, in combination with other NTD programs, other diseases, or other health programs. Substantial heterogeneity existed with respect to the NTD control programs, and no country had integrated all of their NTD control efforts into a single program. NTDs that can be treated with preventative chemotherapy were frequently integrated into a single program. Leprosy control was also frequently integrated with those of other communicable diseases, and notably tuberculosis. Barriers to NTD integration may result from internal administrative obstacles or external obstacles.

**Conclusions:**

Although many countries have begun to integrate NTD control efforts, additional work will be required to realize the full benefits of integration in most of the countries examined here. Moving forward, NTD integration efforts must ensure that administrative structures are designed to maximize the potential success of integrated programs and account for existing administrative processes.

## Author summary

Neglected tropical diseases (NTDs) are communicable diseases that impact billions of people but receive disproportionately little attention from researchers and funders. Many of these diseases have similarities in their epidemiology and control measures, rendering the integration of control programs a practical option to improve accountability, efficiency, and cost-effectiveness. Efforts to integrate NTD control programs have begun across many of the countries with the highest overall burden of NTDs, although no standardized approach for integration exists. Our research sought to examine the landscape of approaches for NTD integration, across the 25 countries with the highest burden of NTDs, to identify models that could be used for countries seeking to integrate their NTD programs. Integration often first targets diseases that can be treated with preventative chemotherapy, though multiple administrative pathways and models exist, including integrating NTD control programs with other NTDs, other communicable diseases, or other health initiatives. Still, no country has yet fully integrated all of their NTD control efforts into a single program. This may be due to internal and external factors that impede the integration of NTD control. Future NTD integration efforts must account for these factors to maximize the potential success of integrated programs.

## Introduction

Neglected tropical diseases (NTDs) are a collection of infectious diseases caused by parasites, viruses, and bacteria. These diseases affect approximately one billion of the world’s poorest people, and most often impact populations living in sub-tropical climates with inadequate access to health care, clean water, sanitation, housing, education and information [1]. All low-income countries are affected by at least five NTDs simultaneously, and 149 countries are affected by at least one NTD [2]. Other estimates suggest that NTDs are some of the world’s most common conditions, accounting for greater than 2 billion infections globally [3]. Countries have worked to combat the impacts of NTDs by implementing “vertical” programs, aimed at preventing specific diseases using top-down approaches. Vertical programs, which are often supported by external funders and organizations, help countries measure success by implementing treatments and lowering prevalence levels. Because of the high burden of NTDs, having successful control programs in place is necessary for decreasing their prevalence and associated morbidity.

Many NTDs have similarities in treatment measures, epidemiology, and geographic distribution [4]. Accordingly, many NTDs have similar strategies for control and eradication. Among the 15 most common NTDs, seven are controlled using preventative chemotherapy in NTD endemic countries [5]. Traditional approaches to NTD control often relied on the aforementioned vertical programs within these countries working in parallel to one another, using the same treatments in the same areas and populations [6]. As a result, although vertical control programs are effective tools in combating specific diseases, integrated disease control programs could enhance control efforts by combining efforts to control multiple diseases into a single intervention.

WHO now recognizes the integration of NTD efforts as a crucial activity for tracking progress, ensuring accountability, and informing the development of policies and strategies [7]. It is in this context that NTD control programs may be incorporated into broader public health systems providing opportunities for countries to advance their NTD control by increasing efficiency, improving the overall quality of health services, covering a larger percentage of the population, and reducing the disparities associated with control programs [8]. Recent disease integration efforts have also yielded considerable savings both financially and in personnel time [9], and modeling efforts have identified opportunities for epidemiological benefits at a population level under some conditions [10]. Thus, the positive impacts of large-scale integrated disease control programs–both for the burden of NTDs, as well as the cost-effectiveness of interventions–may render them the best option for many countries [6]. However, there is no standardized approach to integration, allowing for substantial heterogeneity at the country-level in the implementation, administration, and oversight of integration efforts.

Generally, integrated disease control efforts are administratively placed within Ministries of Health (MOH), and thus the leadership, management, and organizational structures of the ministry can impact the ability to integrate programs. The goal of this work was to understand and present the various was by which NTD endemic countries have approached the integration of NTD control from an administrative standpoint. By observing the different approaches taken by NTD-endemic countries, we hoped to be able to extract common elements which might serve as recommendations or lessons learned that could be provided as a model to other countries that have yet to integrate their NTD control programs.

## Methods

We sought to identify countries most impacted by NTDs, and then selected a sub-set of nations to examine the structure of their respective MOHs, specifically looking at the units and programs overseeing NTD control, and the extent to which they have been integrated. To accomplish this, we used a mixed methods approach that combined a literature review that assessed published evidence on the administrative integration of NTD control efforts, followed with purposely selected key informant interviews to validate the review results and provide additional information not captured in the literature.

### Country selection

To narrow the scope of the study from the almost 150 countries affected by at least one NTD, we chose to focus on 25 countries with multiple endemic NTDs. To do so, we assembled two lists of countries affected by NTDs. The first list examined all countries of the world for the presence of all priority NTDs as defined by the World Health Organization (WHO) (Table 1) [11], focusing on data from 2010 onwards. Using primarily the information provided on WHO’s NTD-specific websites (accessed in 2017 and 2018), which cover priority NTDs and where they are found globally, each country was “scored” for each NTD: 0 indicated a disease was not present within a country; 0.5 indicated that a disease was present within a country but not endemic; and 1 indicated that a country was endemic for a disease. For this work, “endemic” was defined as regular or established occurrence within the boundaries of the country, while “presence” was defined as any reported occurrence. The overall NTD burden was then calculated by totaling the numbers for each country, and the 25 countries with the highest burden of, as measured by our weighted scoring of number of disease presence and endemicity, were identified.

**Table 1:** A comparison of the priority neglected tropical diseases included in the London Declaration’s portfolio and those included in the World Health Organization’sportfolio.

London Declaration NTD Portfolio	WHO NTD Portfolio
	Buruli ulcer
Chagas disease	Chagas disease
	Dengue and Chikungunya
Dracunculiasis	Dracunculiasis
	Echinococcosis
	Foodborne trematodiases
Human African trypanosomiasis	Human African trypanosomiasis
Leishmaniasis	Leishmaniasis
Leprosy	Leprosy
Lymphatic filariasis	Lymphatic filariasis
	Mycetoma, chromoblastomycosis and other deep mycoses
Onchocerciasis	Onchocerciasis
	Rabies
	Scabies and other ectoparasites
Schistosomiasis	Schistosomiasis
Soil-transmitted helminthiases	Soil-transmitted helminthiases
	Snakebite envenoming
	Taeniasis/Cysticercosis
Trachoma	Trachoma

The second list repeated this scoring system, again across all countries in the world, but only using NTDs included within the London Declaration: Chagas disease, dracunculiasis, human African trypanosomiasis, leishmaniasis, leprosy, lymphatic filariasis (LF), onchocerciasis, schistosomiasis, soil-transmitted helminths (STH), and trachoma [12] (Table 1). This was done in efforts to align our analysis towards those countries with the greatest burden of London. Declaration NTDs, which are those prioritized for control and elimination, and are more likely to have existing control initiatives in endemic countries.

The two lists were then reviewed side by side to create a consensus list of the 25 countries with the highest overall burden of NTDs, which we then used to focus our literature review on the types of NTD control programs and approaches. By combining these lists, we sought to expand the geographic scope of the countries reviewed while maintaining a focus on priority NTDs.

### Literature review

We conducted a systematic literature review to characterize the nature of NTD control programs (vertical or integrated) in each of the 25 countries of interest. The review included a broad range of materials, including academic journals, published reports, “grey” literature and other publicly available guidance documents. MOH websites of the 25 countries were reviewed for relevant information on NTD integration efforts. Databases–including Google Scholar, JSTOR, and PubMed–were also searched for materials identifying NTD control programs in the countries of interest. Searches were performed by combining the name of a country and the term “NTD control program.” See S1 Appendix for the complete search strategy. Snowball sampling techniques [13] were used when reviewing these materials to identify other stakeholders involved in NTD integration. The websites of identified stakeholders were also reviewed for information about NTD integration efforts.

Eligibility for inclusion required items to focus on the integration of an NTD control program in a country of interest and to be published in the year 2000 or later. Language restrictions required documents to be written in English or French. No limitations were placed on publication type. This approach was justified to provide a thorough review of information relating the integration of NTD control efforts.

One author performed the initial search, screening of materials, review of full texts, and extraction of data; the search and subsequent screening, review, and extraction was re-performed by a second author to validate and corroborate results. All extracted data was reviewed by a third author for final validation; in cases of discrepancies with data characterization, the data sources were reviewed again and discussed among the remaining authors to reach a consensus. Data extracted characterized NTD control programs as either vertical or integrated. For the purposes of this paper, a program was considered vertical if it focused on a single, specific NTD; programs were considered to be integrated if they combined disease prevention efforts for two or more diseases or conditions (but not necessarily NTDs). In the event that is was unclear if integration had occurred, control programs were assumed to be vertical. If comprehensive control programs were not in place, we characterized programs based on if mapping or surveillance activities had occurred, as a likely precursor to establishment of a control program. Integrated control programs were further characterized based on what other diseases or health programs were integrated with the NTD(s).

### Key informant interviews

Six countries (Brazil, Guinea, India, Kenya, Mali, and Mexico) were selected for further research. These countries were purposively selected to present a range in terms of country size and geography, diversity of endemic NTDs, and in some cases because of existing connections between the researchers and their respective MOH. Individuals from the MOH of these countries were contacted to identify one or more appropriate key informants, validate results from the literature review and gather additional data, particularly related to the opportunities and obstacles for integration of NTD control from an administrative perspective. For all six countries, semi-structured, open-ended interviews were conducted with one or more officials affiliated with NTD control programs within the country and served to highlight key challenges and opportunities with respect to integration efforts. In Mali and Guinea, in addition to MOH personnel, we interviewed officials from non-governmental organizations who collaborate with the MOH to implement national NTD control programs, and who were referred us by their MOH counterparts. A copy of the interview questions sheet used to conduct the semi-structured interviews is provided as Supporting Information.

## Results

### Country selection

**Fig 1:**
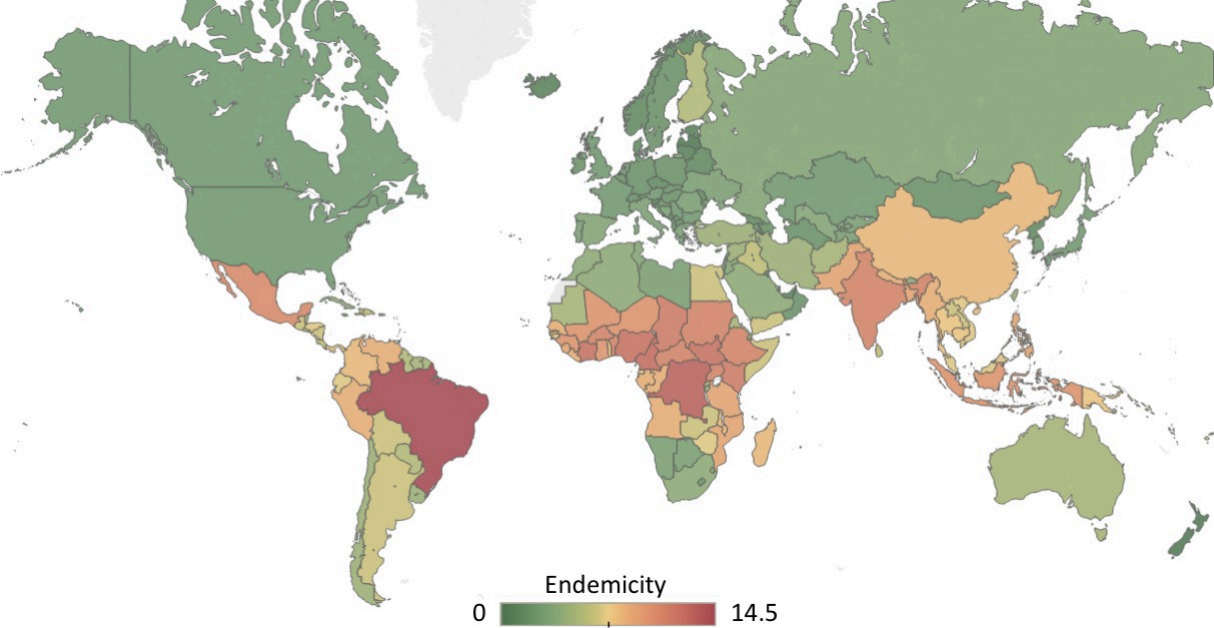
Global burden of NTDs. Twenty NTDs (as defined by the WHO) were assessed in each country to see whether the disease was absent (scored 0), present (scored 0.5), or endemic (scored 1). These values were totaled to calculate the burden, and scores ranged from 0–14.5. Figure created using Tableau Desktop (Seattle, USA) using a base map licensed under the Open Data Commons Open Database License by the Open Street Map Foundation, copyright OpenStreetMap contributors.

The countries with the highest total burden of NTDs were Benin, Brazil, Burkina Faso, Cameroon, Central African Republic, Chad, Côte d’Ivoire, Democratic Republic of the Congo, Ethiopia, Ghana, Guinea, Guinea-Bissau, India, Indonesia, Kenya, Malawi, Mali, Mexico, Mozambique, Niger, Nigeria, South Sudan, Sudan, Tanzania, and Uganda. Geographically, 21 of these countries are located on the African continent, two are located in Asia, one in North America, and one in South America (Fig 1).

### Literature review

Results from the literature review suggest that the integration of NTD programs can be categorized into three groups–full integration of NTD programs, integration of select NTD programs with other NTD programs, and integration of select NTD programs with other public health programs or initiatives.

Substantial heterogeneity existed with respect to the NTD control programs in place. No country had integrated all of their NTD control efforts into a single program. Several countries (Central African Republic, Chad, Guinea, Guinea-Bissau, Indonesia, Mexico and South Sudan) had NTD control profiles that were characterized, per the information available to us, almost exclusively by vertical programs. Of the countries included in this study, Guinea-Bissau’s NTD control efforts were the least robust, as the country lacked control programs for three of the seven NTDs affecting the country.

Chagas disease was the NTD with the fewest programs in place, but it is also the NTD with the smallest geographic distribution. Both countries on our list that are endemic for Chagas–Brazil and Mexico–have control programs in place for the disease [14, 15]. Human African Trypanosomiasis (HAT) was the most geographically widespread disease that had the fewest control programs in place. The disease is present in 19 of the 25 countries considered in this study, but we were unable to find evidence of control programs in three countries–Guinea-Bissau, Niger, and Tanzania–and Ghana and Nigeria only have surveillance programs in place. Leishmaniasis is another disease that often lacks control programs. Leishmaniasis is considered present in Burkina Faso, Ghana, and Nigeria, but based on our literature review, do not currently have leishmaniasis control programs.

The administrative approaches used to integrate NTD programs ranged from multiple NTD combinations, to integrating NTD control with other communicable diseases, to integrating NTD control with Water, Sanitation and Hygiene (WASH) programs (Table 2). MOHs have most frequently integrated control efforts based on similarities in treatment, and the integration of schistosomiasis and STH programs was the most common integration effort. 11 countries–Benin, Brazil, Burkina Faso, Cameroon, Ghana, Kenya, Malawi, Mali, Nigeria, Tanzania, and Uganda–have further integrated schistosomiasis and STH efforts with those for lymphatic filariasis, onchocerciasis, and trachoma [16–22]. Control of these five diseases can be achieved using preventative chemotherapy. Leprosy control efforts were also frequently integrated with those of other communicable diseases caused by *Mycobacterium* species. Ethiopia [23], Tanzania [24], and Uganda [25] have integrated leprosy and tuberculosis (TB) control efforts, Benin has integrated leprosy and Buruli ulcer (BU) [26], Nigeria has integrated leprosy, BU, and TB control efforts [26], and Cameroon has integrated leprosy control efforts with those for BU, yaws, and leishmaniasis [27]. Ethiopia [23], India [28] and Sudan [29] have worked to integrate NTD control efforts, and particularly those with an arthropod vector, with other vector-borne diseases. The integration of NTD and WASH programs has occurred in Cote d’Ivoire (focused on dracunculiasis elimination) [30] and Ethiopia (trachoma) [23]. See S1 Data for a full summary of NTD control programs.

**Table 2:** The integrated control programs involving at least one NTD, for the 25 countries with highest overall NTD burden, based on identified published reports and publicly available data.

NTD Integrated Programs	Countries
5 NTDs	Benin, Brazil^a^, Burkina Faso, Cameroon, Ghana, Kenya, Malawi, Mali, Nigeria, Tanzania, Uganda
4 NTDs	Democratic Republic of the Congo, Niger
3 NTDs	Mozambique
2 NTDs	Cameroon^b^, Cote d’Ivoire, Ethiopia
Leprosy & Tuberculosis	Ethiopia, Nigeria^c^, Tanzania, Uganda
NTDs & vector borne disease	Ethiopia, India, Sudan
NTDs & WASH	Cote d’Ivoire, Ethiopia
NTD & other diseases	Benin^d^

^a^ Brazil’s plan integrates six NTDs—leprosy, lymphatic filariasis, onchocerciasis, schistosomiasis, STH, and trachoma

^b^ Cameroon’s leprosy program is integrated with BU, yaws, and leishmaniasis control efforts

^c^ Nigeria’s leprosy program is integrated with BU, and TB control efforts

^d^ Benin’s leprosy program is integrated with its BU control efforts

For some countries, we identified the existence of a plan, but were unable to verify the details or implementation of NTD control programs. For other countries, sources presented conflicting data. In Tanzania, the Health Sector Strategic Plan for 2015–2020 states that the country will work to improve the detection and management of HAT but does not specifically mention a control program [31]. However, according to WHO, HAT is a priority disease for control and elimination in Tanzania and a focal point exists who is responsible for the coordination and control of activities, which are integrated into an NTD master plan and a national One Health Strategy [32]. A 2010 report [33] suggested that Chad had a schistosomiasis program in place, but more recent documents suggest that no control programs currently exist [34, 35]. Similarly, a 2016 WHO document [36] states that mass drug administration (MDA) efforts for LF have not yet started in South Sudan, but the 2016 South Sudan NTD Plan [37] indicates that MDA is occurring in all parts of the country affected by LF.

**Fig 2:**
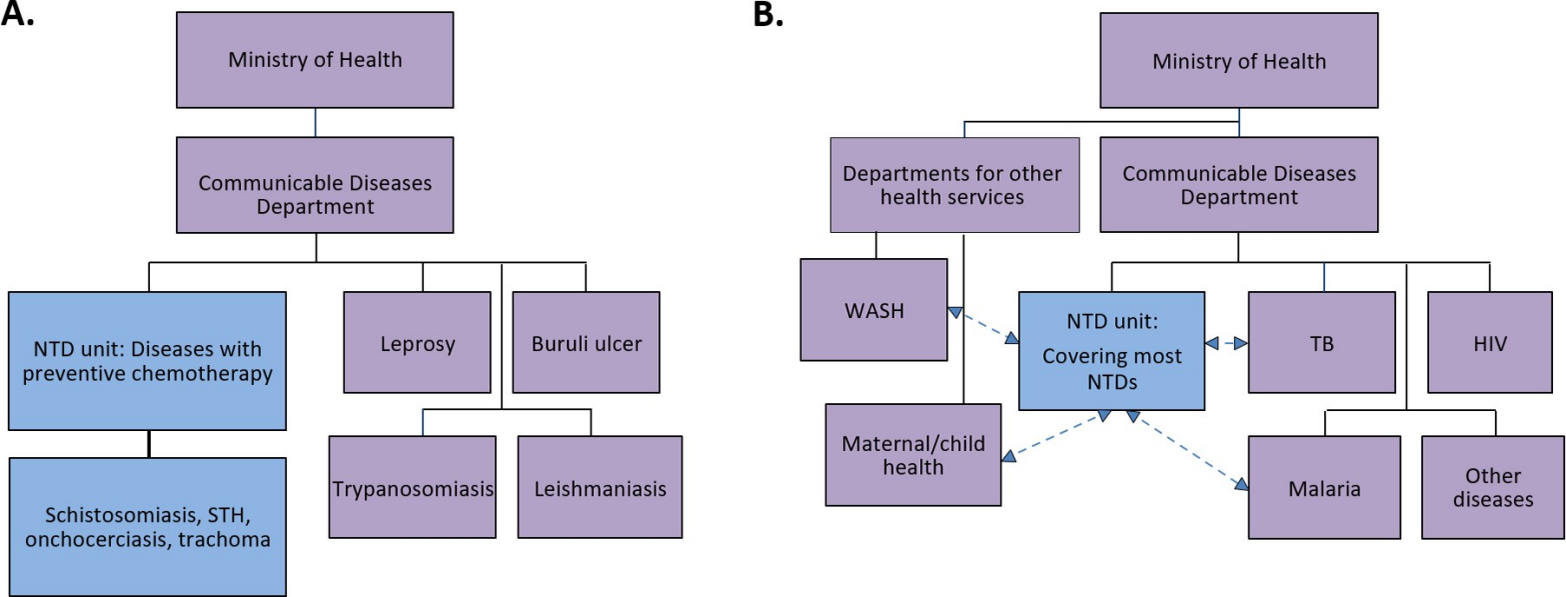
Schematic examples of different administrative organizational approaches to integration of NTD control programs. 2A demonstrates a common approach whereby NTDs that are managed through preventive chemotherapy are integrated administratively, while other NTD programs remain vertically managed. 2B demonstrates a more fully integrated approach, with an “NTD unit” covering most NTD programs, and allowing for linkages with other communicable diseases and/or health services.

**Fig 3:**
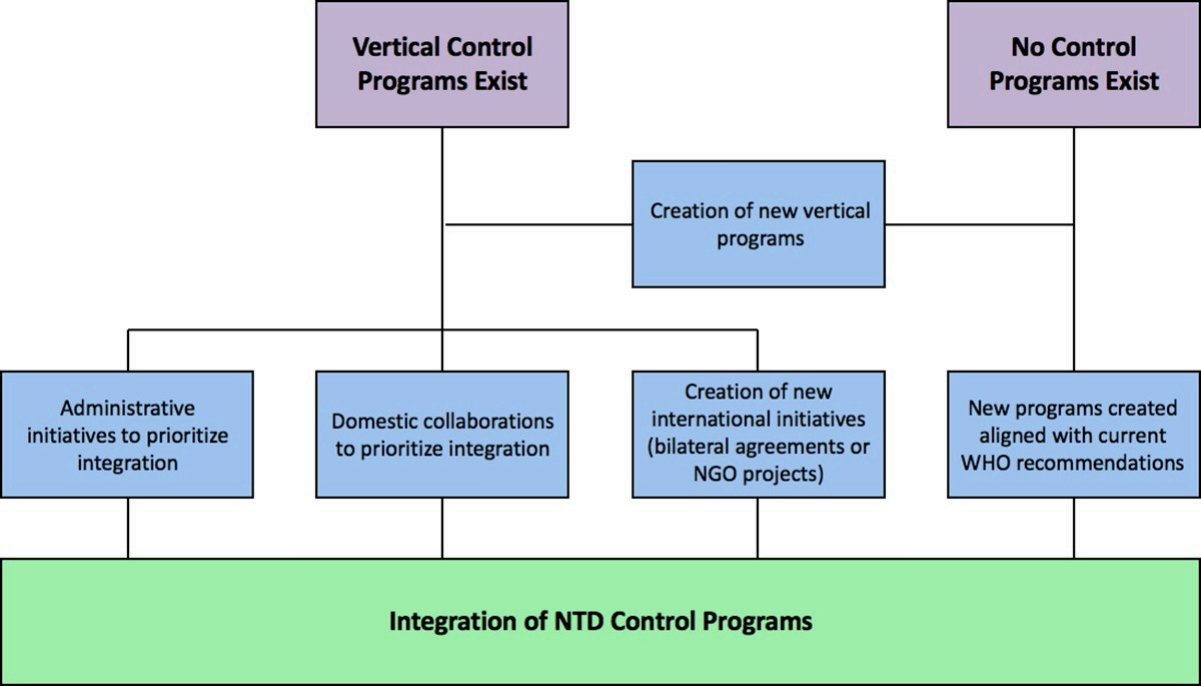
Pathways towards the integration of NTD control programs. These pathways are not mutually exclusive and represent a spectrum of opportunities that could be leveraged to promote effective, integrated NTD control.

Results from the literature review and key informant interviews also revealed important considerations for administration and management of integrated NTD control efforts. Multiple models (Fig 2) and pathways (Fig 3) exist for how integrated programs can be managed by MOHs. Models for integrated NTD programs may span from no administrative integration, with formal or informal coordination between vertically organized units, to partial administrative re-structuring for integration (usually treatment-oriented) (Fig 2A) to more comprehensively integrated units that strive to address all NTDs (Fig 2B), and create linkages with other communicable diseases and/or health services. Several unique pathways exist for transitioning to the integration NTD control programs (Fig 3). Thus far, integration efforts have included the creation of new, integrated MOH-endorsed programs, adjusting administrative structures to expand integrated control efforts, and intersectoral cross-over and collaborations within countries. The creation of new international initiatives and partnerships also represents a pathway towards the integration of NTD control programs.

Key informant interviews revealed that advantages of more fully integrated models included easier oversight of resources and timing of interventions, and stronger advocacy with Ministry leadership for continued integration. This advocacy is important as high-level decision makers within Ministries may not fully recognize the benefits of integration and thus not provide support for the type of administrative restructuring necessary for successful integration. Finally, there are notable administrative obstacles outside of Ministries’ control such as silo-ed funding streams and implementation partners that may, intentionally or not, make the integration of NTD efforts difficult. Interviews also revealed that even if a MOH is willing and able to integrate NTD control programs, earmarked aid from development partners or the organizational structure of implementing partners may prevent them from doing so.

## Discussion

This work highlights the distinct and varied approaches taken by different countries when integrating NTD control programs. Countries with at least one NTD control program frequently have multiple programs in place, often based on funding from non-governmental organizations and public-private partnerships for NTDs focused on vertical elimination and integrated control [6]. All countries included in this study had at least one vertical control program and many of these programs are supported by international development partners–such as the United States Agency for International Development (USAID), the United Kingdom’s Department for International Development (DFID), the World Bank, and the Bill & Melinda Gates Foundation–who can greatly influence the formation and implementation of program activities. Although implementation plans are largely collaborative and country-driven, disease or outcome-targeted funding may also affect opportunities for integration even if the country desires it, discussed in more detail below.

WHO has recently emphasized the need for the integration of vector management, treatment management, information systems, and sectoral collaboration [7] and the data gathered in this study demonstrate that countries are embracing the toward this recommendation of more integrated approaches. This represents a significant step forward for improving health outcomes and the cost-effectiveness of control strategies [8, 9]. Although integration allows for greater control over the allocation of resources, monitoring and evaluation of programs, and other critical activities, these programs also present additional challenges such as greater dependence on the political environment, such as requiring more will, leadership, and long-term resource investment.

Based on our findings, diseases requiring preventive chemotherapy are often first to be integrated administratively, while it is less common for NTDs that require individual case management. This is intuitive, as case management treatment options vary widely for NTDs and may not easily be combined; with lower prevalence levels overall, the incidence of co-infection tends to be lower for these diseases and thus there is reduced opportunity for synergizing patient care for multiple infections. However, given the intensive patient care required for treatment in these cases, there may be opportunities for integration of surveillance and disease management with other aspects of healthcare, such as maternal health visits or health promotion activities. In some instances, NTD integration efforts have focused on commonalities in transmission (e.g. integrated vector management) though fewer formal integrated programs exist in this regard, despite the beneficial opportunities such an approach might provide. One example of this is the *Global Vector Control Response 2017–2030*, endorsed at the 2017 World Health Assembly [38], which has focused on strengthening the prevention of NTDs through inter- and intra-sectoral action and collaboration, and expanding and integrating of vector-control tools and approaches [7]. This strategy also promotes the integration of NTD treatment through MDA campaigns or case management [7]. These actions ultimately should act to increase cost efficiency and help to expand the coverage and sustainability of NTD control efforts.

In accordance with one of their key recommendations of improved integrated surveillance and information systems, WHO has led the development of an integrated NTD database to improve planning and the management of NTD programs allowing for a central, single source of data concerning NTD programs, incorporating input and support from a large number of partners [39]. This platform provides key data on NTDs with the intent of leading to earlier detection of outbreaks [7]. Our literature review and key informant interviews did not uncover evidence that this database is being used by in-country stakeholders for control program planning, so concerted efforts to raise awareness about the availability of this resource may be beneficial. It is also worth noting that the utility of database could be improved by incorporating information regarding control efforts into a single, publicly available platform. This would help to clarify uncertainties surrounding NTD control efforts (e.g., Indonesia, Chad, South Sudan), add a higher level of validity to national NTD control programs through a WHO endorsement, and provide countries opportunities to share lessons learned and best practices with regard to NTD control.

While countries are making substantial progress with regard to conceptualizing NTD control programs, more work is needed. Partnerships between international organizations and national administrative structures may have a role to play in expanding NTD control, as several examples exist whereby vertical programs have been implemented across various countries [40, 41]. Cross-border and regional approaches may have further advantages, particularly where there are high levels of human movement across political boundaries, and allowing countries to benefit from economies of scale for implementation. The Central African Republic, Chad, and Sudan may be especially good candidates for these partnerships as all three of these countries are in close geographic proximity, have similar NTD burdens, and current NTD control efforts are dominated by vertical programs. This regional approach for integration represents one iteration of creating new international initiatives as a pathway for achieving integrated NTD control programs (Fig 3).

Other non-administrative routes may also exist for the integration of NTD control. India, for example, has several NTD control programs, but only leishmaniasis and LF control are integrated under the National Vector Borne Disease Control Programme. Still, in reality, the implementation of these control efforts is conducted by the same personnel who conduct control efforts for other NTDs such as leprosy and STH. Thus, although only two of these NTDs are formally integrated at a higher administrative level, the realities of implementation at a local or community level may nonetheless result in close coordination between control efforts, and thus mirror an integrated approach.

It is also important to consider potential obstacles for NTD control programs, as various factors can influence the delivery of health services. For example, geographical demands, poverty numbers and distribution, resource limitations, and political dynamics can all affect service delivery [42]. For the Central African Republic, Chad, and Sudan, underlying contextual factors may further determine the ability or inability to integrate disease control programs. For example, these countries have all suffered significant instability and civil unrest in recent years. Not only does this civil strife fuel the spread of NTDs–as it is difficult to implement NTD control programs in conflict zones and other non-permissive environments–but it may also deter foreign assistance [43]. Foreign assistance programs may be less willing to implement activities in zones with perceived security risks, or where there is a high chance of interruption of the program due to a renewal of conflict. There may be other restrictions placed on program implementation; for example, the US embargo on Sudan restricted the provision of certain medical equipment and supplies [44]. The sanctions may also have discouraged partners who were concerned with falling afoul of US law. The lifting of sanctions in 2017 [45] points to an opportunity to re-enlist US-based organizations to support NTD control in Sudan and integrate efforts with other priority public health activities.

Effective, integrated responses will also require improved intersectoral collaboration. Brazil launched their national integrated neglected tropical disease plan in 2012 and linked it to the national plan for poverty reduction. In doing so, the country formalized the links between poverty and NTD, which have facilitated implementing effective cross-sector approaches [46]. In another example, India has integrated control programs for soil-transmitted helminths with school health and nutrition programs. This intersectoral collaboration between health and education has acted to expand the reach of NTD programs–improving the health of children across the country [46]. NTD programs have also garnered strong global support–spurring partnerships between governments in NTD endemic countries, international agencies, pharmaceutical companies, international nongovernmental organizations, academia, civil society and United Nations agencies [7]. These collaborations must continue for the full benefits of NTD integration to be realized.

Silo-ed funding streams and the organizational structure of implementation partners may also pose challenges for the integration of NTD control efforts. Earmarked aid from development partners or the organizational structure of implementers may prevent countries from integrating efforts even if they wish to do so. This practice represents a clear divergence from the 2005 Paris Declaration on Aid Effectiveness, which lists alignment as one of the five fundamental principles for making aid more effective [47]. At a more theoretical level, reliance on international aid may also threaten the long-term sustainability of NTD control efforts, in cases where the implemented programs are donor-driven and do not promote country ownership. This could result in a situation in which progress toward integration achieved by MOHs is nullified in the event that development aid is withdrawn.

This study is subject to limitations. Although the countries considered spanned a large geographical range, most were located on the African continent–specifically in sub-Saharan Africa–which may limit the broader applicability of the findings. As our literature review was limited to online sources in English and French, it is possible that we missed information about programs that have been published in other languages, only available in hard copy, or not publicly available, which may have resulted in publication bias. The purposive sampling of personnel involved in NTD control, used for the key informant interviews, may have also biased results, although the primary objective of these interviews was to validate results from the literature review as opposed to primary data collection. Despite these limitations, the results and subsequent discussion presented in this study undoubtedly contribute to a better understanding of the administrative frameworks utilized for the integration of NTD programs.

Moving forward, it is of the utmost importance for advocates of NTD integration to clearly articulate the potential monetary and resource benefits of integration to high-level decision makers to garner political support. These advocates must also take into account the existing administrative structures and creatively engage them to manage the coordination of NTD programs. Finally, it is imperative for development partners to recognize the importance of NTD integration and align their own priorities with national or regional NTD integration efforts where appropriate. In parallel, research efforts should continue to analyze the successes and challenges of integration of disease control programs, in order to produce a robust evidence-base that can support additional refinement of standards and recommendations for future integration.

## Supporting information

S1 Appendix Literature review strategy: Search strategies used for PubMed, JSTOR, and Google Scholar. (DOCX)

S1 Data NTD control programs in countries of interest: Data extraction from literature review. (XLSX)

S1 Fig PRISMA flow diagram: PRISMA flow diagram depicting search strategy and results. (DOC)
