# Supplementary material for: Organization of oversight for integrated control of neglected tropical diseases within Ministries of Health
Source: PLoS Negl Trop Dis. 2018 Nov 21;12(11):e0006929. doi: 10.1371/journal.pntd.0006929 (PMC6281257; doi:10.1371/journal.pntd.0006929)
Supplement: S1 Appendix — (DOCX) [file pntd.0006929.s001.docx]

| ***DATABASE*** | ***SEARCH SYNTAX*** |
| --- | --- |
| PubMed  Date Searched: February 1, 2018 | (Benin OR Brazil OR Burkina Faso OR Cameroon OR Central African Republic OR Chad OR Cote d'Ivoire OR Democratic Republic of the Congo OR Ethiopia OR Ghana OR Guinea OR Guinea-Bissau OR India OR Indonesia OR Kenya OR Malawi OR Mali OR Mexico OR Mozambique OR Niger OR Nigeria OR South Sudan OR Sudan OR Tanzania OR Uganda) AND (“Chagas disease” OR Chagas OR “Dracunculiasis” OR “Human African trypanosomiasis” OR “Leishmaniasis” OR “Leprosy” OR “Lymphatic filariasis” OR “Onchocerciasis” OR “Schistosomiasis” OR “Soil-transmitted helminths” OR “soil-transmitted helminthiasis” OR “Trachoma”) AND (“control program”) |
| JSTOR  Date Searched: February 1, 2018  Country: Benin | (Benin) AND (“Chagas disease” OR Chagas OR “Dracunculiasis” OR “Human African trypanosomiasis” OR “Leishmaniasis” OR “Leprosy” OR “Lymphatic filariasis” OR “Onchocerciasis” OR “Schistosomiasis” OR “Soil-transmitted helminths” OR “soil-transmitted helminthiasis” OR “Trachoma”) AND (“control program”) |
| JSTOR  Date Searched: February 1, 2018  Country: Brazil | (Brazil) AND (“Chagas disease” OR Chagas OR “Dracunculiasis” OR “Human African trypanosomiasis” OR “Leishmaniasis” OR “Leprosy” OR “Lymphatic filariasis” OR “Onchocerciasis” OR “Schistosomiasis” OR “Soil-transmitted helminths” OR “soil-transmitted helminthiasis” OR “Trachoma”) AND (“control program”) |
| JSTOR  Date Searched: February 1, 2018  Country: Burkina Faso | (Burkina Faso) AND (“Chagas disease” OR Chagas OR “Dracunculiasis” OR “Human African trypanosomiasis” OR “Leishmaniasis” OR “Leprosy” OR “Lymphatic filariasis” OR “Onchocerciasis” OR “Schistosomiasis” OR “Soil-transmitted helminths” OR “soil-transmitted helminthiasis” OR “Trachoma”) AND (“control program”) |
| JSTOR  Date Searched: February 1, 2018  Country: Cameroon | (Cameroon) AND (“Chagas disease” OR Chagas OR “Dracunculiasis” OR “Human African trypanosomiasis” OR “Leishmaniasis” OR “Leprosy” OR “Lymphatic filariasis” OR “Onchocerciasis” OR “Schistosomiasis” OR “Soil-transmitted helminths” OR “soil-transmitted helminthiasis” OR “Trachoma”) AND (“control program”) |
| JSTOR  Date Searched: February 1, 2018  Country: Central African Republic | (Central African Republic) AND (“Chagas disease” OR Chagas OR “Dracunculiasis” OR “Human African trypanosomiasis” OR “Leishmaniasis” OR “Leprosy” OR “Lymphatic filariasis” OR “Onchocerciasis” OR “Schistosomiasis” OR “Soil-transmitted helminths” OR “soil-transmitted helminthiasis” OR “Trachoma”) AND (“control program”) |
| JSTOR  Date Searched: February 1, 2018  Country: Chad | (Chad) AND (“Chagas disease” OR Chagas OR “Dracunculiasis” OR “Human African trypanosomiasis” OR “Leishmaniasis” OR “Leprosy” OR “Lymphatic filariasis” OR “Onchocerciasis” OR “Schistosomiasis” OR “Soil-transmitted helminths” OR “soil-transmitted helminthiasis” OR “Trachoma”) AND (“control program”) |
| JSTOR  Date Searched: February 1, 2018  Country: Cote d’Ivoire | (Cote d'Ivoire) AND (“Chagas disease” OR Chagas OR “Dracunculiasis” OR “Human African trypanosomiasis” OR “Leishmaniasis” OR “Leprosy” OR “Lymphatic filariasis” OR “Onchocerciasis” OR “Schistosomiasis” OR “Soil-transmitted helminths” OR “soil-transmitted helminthiasis” OR “Trachoma”) AND (“control program”) |
| JSTOR  Date Searched: February 1, 2018  Country: Democratic Republic of the Congo | (Democratic Republic of the Congo) AND (“Chagas disease” OR Chagas OR “Dracunculiasis” OR “Human African trypanosomiasis” OR “Leishmaniasis” OR “Leprosy” OR “Lymphatic filariasis” OR “Onchocerciasis” OR “Schistosomiasis” OR “Soil-transmitted helminths” OR “soil-transmitted helminthiasis” OR “Trachoma”) AND (“control program”) |
| JSTOR  Date Searched: February 1, 2018  Country: Ethiopia | (Ethiopia) AND (“Chagas disease” OR Chagas OR “Dracunculiasis” OR “Human African trypanosomiasis” OR “Leishmaniasis” OR “Leprosy” OR “Lymphatic filariasis” OR “Onchocerciasis” OR “Schistosomiasis” OR “Soil-transmitted helminths” OR “soil-transmitted helminthiasis” OR “Trachoma”) AND (“control program”) |
| JSTOR  Date Searched: February 1, 2018  Country: Ghana | (Ghana) AND (“Chagas disease” OR Chagas OR “Dracunculiasis” OR “Human African trypanosomiasis” OR “Leishmaniasis” OR “Leprosy” OR “Lymphatic filariasis” OR “Onchocerciasis” OR “Schistosomiasis” OR “Soil-transmitted helminths” OR “soil-transmitted helminthiasis” OR “Trachoma”) AND (“control program”) |
| JSTOR  Date Searched: February 1, 2018  Country: Guinea | (Guinea) AND (“Chagas disease” OR Chagas OR “Dracunculiasis” OR “Human African trypanosomiasis” OR “Leishmaniasis” OR “Leprosy” OR “Lymphatic filariasis” OR “Onchocerciasis” OR “Schistosomiasis” OR “Soil-transmitted helminths” OR “soil-transmitted helminthiasis” OR “Trachoma”) AND (“control program”) |
| JSTOR  Date Searched: February 1, 2018  Country: Guinea-Bissau | (Guinea-Bissau) AND (“Chagas disease” OR Chagas OR “Dracunculiasis” OR “Human African trypanosomiasis” OR “Leishmaniasis” OR “Leprosy” OR “Lymphatic filariasis” OR “Onchocerciasis” OR “Schistosomiasis” OR “Soil-transmitted helminths” OR “soil-transmitted helminthiasis” OR “Trachoma”) AND (“control program”) |
| JSTOR  Date Searched: February 1, 2018  Country: India | (India) AND (“Chagas disease” OR Chagas OR “Dracunculiasis” OR “Human African trypanosomiasis” OR “Leishmaniasis” OR “Leprosy” OR “Lymphatic filariasis” OR “Onchocerciasis” OR “Schistosomiasis” OR “Soil-transmitted helminths” OR “soil-transmitted helminthiasis” OR “Trachoma”) AND (“control program”) |
| JSTOR  Date Searched: February 1, 2018  Country: Indonesia | (Indonesia) AND (“Chagas disease” OR Chagas OR “Dracunculiasis” OR “Human African trypanosomiasis” OR “Leishmaniasis” OR “Leprosy” OR “Lymphatic filariasis” OR “Onchocerciasis” OR “Schistosomiasis” OR “Soil-transmitted helminths” OR “soil-transmitted helminthiasis” OR “Trachoma”) AND (“control program”) |
| JSTOR  Date Searched: February 1, 2018  Country: Kenya | (Kenya) AND (“Chagas disease” OR Chagas OR “Dracunculiasis” OR “Human African trypanosomiasis” OR “Leishmaniasis” OR “Leprosy” OR “Lymphatic filariasis” OR “Onchocerciasis” OR “Schistosomiasis” OR “Soil-transmitted helminths” OR “soil-transmitted helminthiasis” OR “Trachoma”) AND (“control program”) |
| JSTOR  Date Searched: February 1, 2018  Country: Malawi | (Malawi) AND (“Chagas disease” OR Chagas OR “Dracunculiasis” OR “Human African trypanosomiasis” OR “Leishmaniasis” OR “Leprosy” OR “Lymphatic filariasis” OR “Onchocerciasis” OR “Schistosomiasis” OR “Soil-transmitted helminths” OR “soil-transmitted helminthiasis” OR “Trachoma”) AND (“control program”) |
| JSTOR  Date Searched: February 1, 2018  Country: Mali | (Mali) AND (“Chagas disease” OR Chagas OR “Dracunculiasis” OR “Human African trypanosomiasis” OR “Leishmaniasis” OR “Leprosy” OR “Lymphatic filariasis” OR “Onchocerciasis” OR “Schistosomiasis” OR “Soil-transmitted helminths” OR “soil-transmitted helminthiasis” OR “Trachoma”) AND (“control program”) |
| JSTOR  Date Searched: February 1, 2018  Country: Mexico | (Mexico) AND (“Chagas disease” OR Chagas OR “Dracunculiasis” OR “Human African trypanosomiasis” OR “Leishmaniasis” OR “Leprosy” OR “Lymphatic filariasis” OR “Onchocerciasis” OR “Schistosomiasis” OR “Soil-transmitted helminths” OR “soil-transmitted helminthiasis” OR “Trachoma”) AND (“control program”) |
| JSTOR  Date Searched: February 1, 2018  Country: Mozambique | (Mozambique) AND (“Chagas disease” OR Chagas OR “Dracunculiasis” OR “Human African trypanosomiasis” OR “Leishmaniasis” OR “Leprosy” OR “Lymphatic filariasis” OR “Onchocerciasis” OR “Schistosomiasis” OR “Soil-transmitted helminths” OR “soil-transmitted helminthiasis” OR “Trachoma”) AND (“control program”) |
| JSTOR  Date Searched: February 1, 2018  Country: NIger | (Niger) AND (“Chagas disease” OR Chagas OR “Dracunculiasis” OR “Human African trypanosomiasis” OR “Leishmaniasis” OR “Leprosy” OR “Lymphatic filariasis” OR “Onchocerciasis” OR “Schistosomiasis” OR “Soil-transmitted helminths” OR “soil-transmitted helminthiasis” OR “Trachoma”) AND (“control program”) |
| JSTOR  Date Searched: February 1, 2018  Country: Nigeria | (Nigeria) AND (“Chagas disease” OR Chagas OR “Dracunculiasis” OR “Human African trypanosomiasis” OR “Leishmaniasis” OR “Leprosy” OR “Lymphatic filariasis” OR “Onchocerciasis” OR “Schistosomiasis” OR “Soil-transmitted helminths” OR “soil-transmitted helminthiasis” OR “Trachoma”) AND (“control program”) |
| JSTOR  Date Searched: February 1, 2018  Country: South Sudan | (South Sudan) AND (“Chagas disease” OR Chagas OR “Dracunculiasis” OR “Human African trypanosomiasis” OR “Leishmaniasis” OR “Leprosy” OR “Lymphatic filariasis” OR “Onchocerciasis” OR “Schistosomiasis” OR “Soil-transmitted helminths” OR “soil-transmitted helminthiasis” OR “Trachoma”) AND (“control program”) |
| JSTOR  Date Searched: February 1, 2018  Country: Sudan | (Sudan) AND (“Chagas disease” OR Chagas OR “Dracunculiasis” OR “Human African trypanosomiasis” OR “Leishmaniasis” OR “Leprosy” OR “Lymphatic filariasis” OR “Onchocerciasis” OR “Schistosomiasis” OR “Soil-transmitted helminths” OR “soil-transmitted helminthiasis” OR “Trachoma”) AND (“control program”) |
| JSTOR  Date Searched: February 1, 2018  Country: Tanzania | (Tanzania) AND (“Chagas disease” OR Chagas OR “Dracunculiasis” OR “Human African trypanosomiasis” OR “Leishmaniasis” OR “Leprosy” OR “Lymphatic filariasis” OR “Onchocerciasis” OR “Schistosomiasis” OR “Soil-transmitted helminths” OR “soil-transmitted helminthiasis” OR “Trachoma”) AND (“control program”) |
| JSTOR  Date Searched: February 1, 2018  Country: Uganda | (Uganda) AND (“Chagas disease” OR Chagas OR “Dracunculiasis” OR “Human African trypanosomiasis” OR “Leishmaniasis” OR “Leprosy” OR “Lymphatic filariasis” OR “Onchocerciasis” OR “Schistosomiasis” OR “Soil-transmitted helminths” OR “soil-transmitted helminthiasis” OR “Trachoma”) AND (“control program”) |
| Google Scholar  Date Searched: February 3, 2018 | (Benin OR Brazil OR Burkina Faso OR Cameroon OR "Central African Republic" OR Chad OR Cote d'Ivoire OR "Democratic Republic of the Congo" OR Ethiopia OR Ghana OR Guinea OR Guinea-Bissau OR India OR Indonesia OR Kenya OR Malawi OR Mali OR Mexico OR Mozambique OR Niger OR Nigeria OR South Sudan OR Sudan OR Tanzania OR Uganda) AND (“Chagas disease” OR Chagas OR “Dracunculiasis” OR “Human African trypanosomiasis” OR “Leishmaniasis” OR “Leprosy” OR “Lymphatic filariasis” OR “Onchocerciasis” OR “ Schistosomiasis” OR “Soil-transmitted helminths” OR “soil-transmitted helminthiasis” OR “Trachoma”) AND (“control program”) |
