# Supplementary material for: Organization of oversight for integrated control of neglected tropical diseases within Ministries of Health
Source: PLoS Negl Trop Dis. 2018 Nov 21;12(11):e0006929. doi: 10.1371/journal.pntd.0006929 (PMC6281257; doi:10.1371/journal.pntd.0006929)
Supplement: S1 Fig — (DOC) [file pntd.0006929.s003.doc]

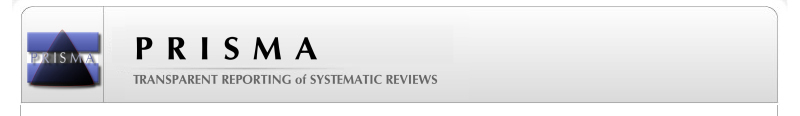
**PRISMA 2009 Flow Diagram**

**Screening**

**Included**

**Eligibility**

**Identification**

Records identified through database searching
(n = 680)

Additional records identified through other sources
(n = 43)

Records after duplicates removed
(n = 723)

Records screened
(n = 723)

Records excluded
(n = 562)

Full-text articles assessed for eligibility
(n = 161)

Full-text articles excluded
(n = 107)

Studies included in analysis
(n = 54)
